# Advances in Gut Microbiota of Viral Hepatitis Cirrhosis

**DOI:** 10.1155/2019/9726786

**Published:** 2019-11-22

**Authors:** Yixuan Wang, Calvin Q. Pan, Huichun Xing

**Affiliations:** ^1^Institute of Infectious Disease, Peking University Ditan Teaching Hospital, Beijing, China; ^2^Division of Gastroenterology and Hepatology, Department of Medicine, NYU Langone Health, New York University School of Medicine, New York, NY, USA; ^3^Department of Hepatology Division 3, Beijing Ditan Hospital, Capital Medical University, Beijing, China

## Abstract

Although gut dysbiosis appears in 20%–75% of cirrhotic patients, there are limited data on microbiota profiles in viral hepatitis cirrhotics and its role in progression to cirrhosis. Further understanding on the relationship between gut dysbiosis and cirrhosis presents a unique opportunity in not only predicting the development of cirrhosis but also discovering new therapies. Recent advances have been made on identifying unique microbiota in viral hepatitis cirrhotics and adopting the microbiota index to predict cirrhosis. Therapeutic intervention with microbiome-modulating has been explored. Cirrhosis from viral infection has unique bacterial or fungal profiles, which include increased numbers of *Prevotella*, *Streptococcus*, Staphylococcaceae, and *Enterococcus*, as well as decreased *Ruminococcus* and *Clostridium*. In addition, the gut microbiota can stimulate liver immunity, effectively helping hepatitis virus clearance. In clinical settings, CDR, GDI, *Basidiomycota*/*Ascomycota*, specific POD, and so forth are efficient microbiota indexes to diagnose or prognosticate cirrhosis from viral hepatitis. FMT, probiotics, and prebiotics can restore microbial diversity in cirrhotic patients with viral hepatitis, decrease ammonia serum or endotoxemia levels, prevent complications, reduce rehospitalization rate, and improve prognosis. Cirrhotics from viral hepatitis had unique bacterial or fungal profiles, associated with specific metabolic, immune, and endocrinological statuses. Such profiles are modifiable with medical treatment. The role of gut archaea and virome, implementation of FMT, microbiota metabolites as adjuvant immunotherapy, and microbiota indexes for prognostication deserve attention.

## 1. Introduction

Viral hepatitis cirrhosis is a chronic liver disease characterized by persistent infection of hepatitis B and C viruses, diffuse fibrosis, and formation of pseudolobule caused by long-term liver injury [1]. Recently, research about gut microbiome has emerged in liver disease fields. Samples are often derived from saliva, mucous, feces, and lavage fluids. A variety of study methods has been applied, including traditional incubation, bacterial 16S ribosomal RNA (rRNA) Illumina MiSeq sequencing, fungal 18S rRNA Illumina MiSeq sequencing, Internal Transcribed Spacer Sequencing, and Shotgun Metagenomic Sequencing [2]. Emerging data suggest a significant role of gut dysbiosis in viral hepatitis cirrhotics. Recent studies have illustrated the unique microbiota profiles in cirrhotics, such as decreased Firmicutes or increased *Bacteroidetes*. The relationship between gut microbiota and liver metabolism, as well as the interaction between gut dysbiosis and the progression of fibrosis, has been explored. Using microbiota indexes such as the Cirrhosis Dysbiosis Ratio or Gut Dysbiosis Index to predict prognosis has been proposed. Furthermore, studies have suggested probiotic and faecal microbiota transplantation (FMT) as microbiome-modulating therapies. For the current review, literature searches were mainly conducted for publications from January 2013 to present in PubMed databases by using the following key words: “gut microbiota”/“gut dysbiosis”/“gut microbiome” + “cirrhosis,” “gut biofilm”/“gut microbiota metabolite”/“gut fungal” + “cirrhosis,” “Gut-Liver Axis”/“PPI”/“Antibiotics”/“probiotics”/“prebiotic”/“FMT” + “cirrhosis”/“microbiota”/“liver.” Among 1149 articles we retrieved, 1086 articles were excluded because there were not for viral hepatitis infection or not related to our topic. Several studies were excluded for duplicated core content which we have summarized. In addition, we included four important articles before 2013. Publications with data on coinfection of HBV and other viral hepatitis were included in our review. Data on gut microbiota in liver cirrhotics were extracted and summarized. Our review is focused on discussing new findings on the aforementioned topics.

## 2. Current Understanding of Gut Microbiota

### 2.1. Gut Microbiota Biofilms and Colonization

The human gut microbiota consists of 10–100 trillion bacteria, fungi, archaea, and viruses, although sometimes the term specifically refers to bacterial communities. Living as exopolysaccharide-coated biofilms that disperse free-swimming bacteria, the gut microbiota resides over the outer layer of the intestinal mucus. These biofilms are capable of retaining water not only to protect against antimicrobial substances and digestive enzymes but also to facilitate quorum sensing and horizontal gene transfer [3]. Once cathepsin protease from enteropathogens such as bacteria, viruses, or parasites disrupts the biofilm exopolysaccharide, it may promote the release of pathobionts, followed by translocating pathobionts through human epithelia paracellularly and transcellularly. As a result, the production of proinflammatory mediators like CXCL-8 and IL-1 is increased [4].

The colonization of the gut microbiota begins in as early as the time of delivery when a baby encounters microorganisms from the birth canal. Soon after birth, more microbes reach the gut though the feeding process as the baby suckles the mother's breast or milk bottle with existing microorganisms colonized in the skin or feeding bottle [5]. Although it is not clear how the baby's first microbes get into the body, some researchers suggest that the first microbial exposure occurs even before delivery because there is a bacterial presence in the placenta, umbilical cord, and amniotic fluid in healthy full-term pregnancies. By the age of two or three years old, the composition of a child's gut microbiota tends to be stable with a variety of flora and is very similar to the profile presented in an adult's gut [6].

### 2.2. Gut Microbiota Metabolites and Their Functions

The liver synthesizes primary bile acids from cholesterol, and conjugated with taurine or glycine, through the classical pathway of 7*α*-hydroxycholesterol and the 27-hydroxycholesterol alternative pathway. Then it releases primary bile acid into the intestine, where secondary bile acid is generated under the action of gut bacteria with bile salt hydrolase, 3*α*-, 7*α*-, 12*α*-hydroxysteroid dehydrogenase, predominantly in the colon, and involves multiple steps including deconjugation, dehydroxylation, epimerization, and oxidation [7]. Bile acid signals molecules via the nuclear farnesoid X receptor and the Takeda G protein coupled receptor 5, affecting lipid and carbohydrate metabolism, energy expenditure, and inflammation [8].

Gut microbiota metabolites are produced by gut microbiota through metabolizing bile acid, aromatic amino acids, carbohydrates, and polysaccharides. These metabolites are mainly secondary bile acid, indole or phenol derivatives, short-chain fatty acids (SCFAs), adenosine triphosphate (ATP), and polysaccharide A.

Tryptophan catabolites include indole, indole-3-propionic acid, indole-3-acetic acid, indole-3-aldehyde, tryptamine, and 3-methylindole. The 3-methylindole promotes the activation of AhR and NR1I2 with strengthening of the integrity of intestinal mucosa and epithelial barrier function, resulting in the reduction of bacteria translocation [9]. Indole-3-propionic acid, a tryptophan metabolite produced by gut bacteria, can inhibit NF-κB signaling and reduces the levels of proinflammatory cytokines to repress hepatic inflammation and liver injury [10]. Other gut microbiota metabolites are classified as phenol derivatives, which are derived from tyrosine. Uchiyama et al. [11] suggested that phenol derivatives had bioactivity of preventing lipopolysaccharide- (LPS-) induced proinflammatory gene expression in the liver of mice. Among SCFAs, three molecules including acetic, propionic, and butyric acids are the major products of carbohydrate fermentation, and the gut microbiota produces 50–100 mM daily of these compounds. Butyrate is a main product of the commensal bacteria, such as genus *Clostridia*, *Faecalibacterium*, and *Roseburia*. Several studies suggest that butyrate serves as an energy substrate for the colonocyte, whereas acetate and propionate are substrates for glucose and fatty acid synthesis [12]. Gut microbiota could generate a large amount of ATP. A recent study by Perruzza et al. [13] suggested that the extracellular bacterially derived ATP limited the secretory IgA response in the small intestine, which may affect the homeostasis of gut commensal bacteria including the quantity and composition. Polysaccharide A as a unique product of gut bacterium *Bacteroides fragilis* was found to be able to upregulate the Toll-like receptor (TLR) 2 expression on the surface of DCs and promote both potential anti-inflammatory cytokine IL-10 secretion from CD4+ T cells and the differentiation of naive CD4+ T cells into Th1 cells over Th2 cells [14].

### 2.3. Gut Microbiota as an Endocrine Organ

The gut microbiota or its metabolites can serve as direct acting compounds or indirectly regulate numerous hormonal chemicals. Several neurotransmitters are affected or regulated through the bioactivities of microbiota and the metabolites, including neuroactive compounds such as serotonin dopamine, noradrenaline, and *γ*-aminobutyric acid; precursors to neuroactive compounds and hypothalamus hormone cortisol; and gastrointestinal hormones. The gastrointestinal hormones are mainly ghrelin, leptin, glucagon-like peptide-1, and the peptide tyrosine [15]. Giving bioactive functions of the gut microbiota and metabolites, they have been considered as a major neurological and endocrine organ. Published studies have demonstrated that certain gut bacteria produce the neurotransmitters of noradrenaline, dopamine, and serotonin, whereas several *Lactobacilli* could generate *γ*-aminobutyric acid. In addition, gut microbiota metabolites like SCFAs regulate the secretion of serotonin and peptide tyrosine which play a major role in the gut-brain axis [16]. The aforementioned hormonal molecules are closely related to the human metabolism, stress response, and neurological function. However, their roles in disease state have not been fully explored yet. Hormonal chemicals also have effects on gut microbiota and the dynamic of bacteria expansion. Freestone et al. [17] reported that noradrenaline stimulates the growth of nonpathogenic commensal *Escherichia coli* and other gram-negative bacteria. Hormonal chemicals like catecholamines are required for the induction of sulfatase activity in *Salmonella* as well as enhancing the virulence of other pathogenic bacteria [18]. The complexity of host-microbiota cross-talk needs to be further investigated and explored in future studies.

## 3. Viral Hepatitis Infection and Gut Microbiota

### 3.1. Immune Response in Viral Hepatitis Cirrhosis

HBV, a partially double-stranded hepatotropic DNA virus, can establish a persistent and chronic infection in humans. HBV invasion process involves viral internalization (HBV interacts with hepatic bile acid transporter sodium taurocholate cotransporting polypeptide), rcDNA converted into closed circular DNA, formation of two strands HBV DNA and nucleocapsids, and exiting the hepatocytes through the secretory pathway [19]. At the initial stages, innate immune response to HBV infection primarily depends on the recognition of Toll-like receptors (TLRs), secretion of type 1 IFN-*α*/*β* cytokines, and activation of NK cells and NKT cells. As the main effectors of HBV clearance, HBV-specific CD4+ and CD8+ T cells induce the production of numerous cytokines and IFN-*γ* antibodies specifically against HBV [20]. HCV, a single-stranded hepatotropic RNA virus, induces a large number of IFN-stimulated genes, dysfunctional CD4+ T cells, and stunned CD8+ T cells. The host immune activation on clearing HBV or HCV may lead to chronic inflammation and necrosis, resulting in progressive fibrosis and the development of liver cirrhosis [21]. Gut microbiota metabolites can both induce and promote host immune response. Gut microbiota-derived butyric acids promote the survival of CD8+ T cells and enhanced memory potential of activated CD8+ T cells through uncoupling the tricarboxylic acid cycle from glycolytic input, as an optimal substance recall immunoreaction upon antigen reencounter [22]. Chou et al. [23] suggested that antibiotic-treated mice experienced an impaired adaptive immunity against HBV; only those with maturation of gut microbiota can stimulate liver immunity effectively, resulting in rapid HBV clearance.

### 3.2. Gut-Liver Axis in Healthy Liver

The portal system, which serves as a highway from the intestine to the liver, can transfer bacteria and their products to the liver and modulate the host immune system, called the “Gut-Liver Axis.” Above all, bile acid enterohepatic circulation plays a vital role in Gut-Liver Interaction, involving bile acid synthesis, detoxification, and transport throughout the Gut-Liver Axis, reabsorbed by the terminal ileum cholangiocytes, colonocytes, and proximal convoluted renal tubules, and finally recycled to the liver through portal system and mainly taken up by NTCP and OATPs. Bile acid released by the gallbladder, through the bile duct and into the intestinal lumen, can directly destroy the bacterial membrane or indirectly generate substances like nitric oxide and IL-18 via the “TBA-TGR5-FXR-cAMP” pathway to affect the gut microbiota [24]. Secretory IgAs are also indispensable in regulating host-microbiota homeostasis. IgA, produced by intrahepatic B-cell Peyer patches against intestinal antigens, agglutinates bacteria and participates in biofilm formation preventing bacterial translocation [7]. Furthermore, high IgA coating uniquely identifies colitogenic intestinal bacteria.

### 3.3. The Impact of Viral Hepatitis Cirrhosis on Gut Microbiota

Hepatic inflammation is always accompanied by low bile acid production and an increase in expression of bile salt transporters. Since bile acids exert a bacteriostatic effect, directly destroying the bacterial membrane or indirectly generating substances like NO and IL-18, especially towards anaerobic bacteria, the 7*α*-dehydroxylating bacterial populations tend to collapse due to low selection pressures. A lower 7*α*-dehydroxylating bacteria representation is associated with a reciprocal expansion of potentially pathogenic *Enterobacteriaceae* [25]. In cirrhotic patients, cholestasis results in portal hypertension and bleeding causes intestinal mucosal edema and ischemia, or even reperfusion injury. Abnormal hepatic vascular function or portal hypertension affects the composition of gut microbiota, maybe due to its altered intestinal motility. Clostridiales and Bacteroidales classes were independently associated with variations in portal vein area and portal flow in cirrhotic rats [25]. The aforementioned pathological changes contribute to the status of intestinal dyskinesia, the retention of intestinal contents, and increased intestinal permeability. Consequently, prompt colonic bacteria migrate to the jejunum and duodenum, resulting in small intestinal bacterial overgrowth (SIBO). Under the dual effects of intestinal permeability and SIBO, intestinal bacteria, other gut microorganisms, and microbial metabolites can pass through the lymphatic system or gut barrier, causing bacterial translocation (which can be evaluated by plasma endotoxin assay), endotoxemia, spontaneous bacterial peritonitis, and so forth [26].

### 3.4. Gut Dysbiosis Mediates the Liver Injury and Progression of Viral Hepatitis Cirrhosis

**Figure 1:**
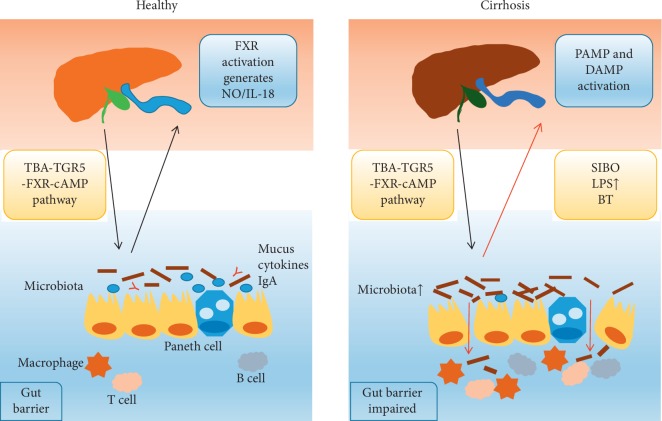
Gut-Liver Interaction in healthy and cirrhosis.

The progression of cirrhosis is associated with increased intestinal permeability, SIBO, and bacterial translocation. Once intestinal microbial metabolites such as ethanol, acetaldehyde, trimethylamine, and short-chain and free fatty acids, bacterial endotoxins, or other substances break the gut barrier and enter into the portal system, the Gut-Liver Axis allows them to attack the liver through the pathogen-associated molecular pattern (PAMP) and damage-associated molecular pattern (DAMP) pathway, activating the liver's immune system and increasing tertiary lymphoid structures (TLS) or nucleotide binding oligomerization domain-like receptor (NLR) activation in the liver with consequential cytokine production, liver inflammation, fibrogenesis, and cirrhosis [27] (Figure 1). A recent study found bacterial-induced (like *E. coli*-induced) resistin production can downregulate the inflammatory response of macrophages and neutrophil function and thus may jeopardize the elimination of bacteria that translocate to ascitic fluid and facilitate bacterial infection [28]. Moreover, increased Alcaligenaceae and Porphyromonadaceae, Veillonellaceae, *Enterococcus*, *Megasphaera*, and *Burkholderia* were related to the high ammonia levels and systemic inflammation and thus worsen the hepatic encephalopathy (HE) symptoms by means of ammoniagenesis and generation of endotoxin-driven inflammatory response (higher IL-6, TNF-*α*, IL-2, and IL-13) [29].

## 4. GUT Profile in Viral Hepatitis Cirrhotics

### 4.1. Gut Bacterial Profile in Viral Hepatitis Cirrhotics

Although the individual gut bacteria are unique at the species level, they are stable at the phylum level. The main phyla are Bacteroides and Firmicutes, followed by Proteobacteria and Actinobacteria. Enterotypes are mostly driven by phylum or genus composition. Enterotype 1 had Bacteroides as its best indicator, is associated with diets enriched in animal proteins and saturated fats, and tends to reduce overall bacterial diversity and significantly increase lymphocyte and C-reactive protein. Enterotype 2 was driven by *Prevotella*, was inversely correlated with Bacteroides, and overall decreased lipolytic proteolytic fermentation potential, implying high risk of chronic inflammatory bowel disease. Enterotype 3 was distinguished by an overrepresentation of Firmicutes and associated with low host-inflammatory response [30].

Recently, Ren et al. [31] enrolled 75 health controls and 40 hepatitis B cirrhotics. Compared with the controls, three faecal microbial diversity indexes (Shannon index, Simpson index, and Invsimpson index) were significantly decreased in cirrhotics. There were 768 OTUs in controls and 632 OTUs in CHB cirrhotics. At the phylum level, compared with the controls, Firmicutes and Fusobacteria of cirrhotics decreased significantly, while Bacteroidetes and Proteobacteria of cirrhosis increased significantly in cirrhotics. At genus level, *Bacteroides* and *Faecalibacterium* decreased, while *Prevotella* and *Escherichia* significantly increased. Moreover, there are numerous studies about gut microbiota of liver diseases which enrolled hepatitis B or hepatitis C cirrhotics. Those studies are summarized in Table 1.

### 4.2. Gut Fungal Profile in Viral Hepatitis Cirrhotics

Guo [39] used the fungal 18SrRNA library to investigate the partial characteristics of gut fungal microbiomes in hepatitis B cirrhotics. The study showed 27 different fungal species belonging to three main classes Ascomycetes (81.5%), Basidiomycetes (14.8%), and Zygomycetes (3.7%). Real-time fluorescent PCR was performed on the most common fungi species and the results revealed that *Candida albicans*, *Candida krusei*, and *Candida glabrata* were significantly increased in the CHB cirrhosis group compared with health controls. Recently, Bajaj et al. [40] enrolled 143 cirrhotics (72 HCV-derived) and 26 health controls. Compared with health controls, the fungal diversity decreased. The relative abundance of *Basidiomycota* decreased, while Ascomycota *Candida* increased. Similar with bacteria diversity, fungal diversity is stable over time and can be changed when using antibiotics or when infection occurs. The study also found that the lower the ratio of fungal Basidiomycota/Ascomycota abundance, the higher the MELD score and severity of cirrhosis, and the lower the ratio of Bacteroides/Ascomycota abundance, the higher the 90-day hospitalization rate, indicating that fungal and bacterial dysbiosis independently impacts the hospitalization risk and therefore both need to be considered.

Besides, cirrhotics have a high rate of fungal infection. A large cohort study which enrolled 2,743 hospitalized cirrhotics (940 HCV-derived) showed that 1,052 cirrhotics (348 HCV-derived) were infected, of which 36 HCV-derived cirrhotics had fungal infection, most of which were *Candida* infection [41]. The latest research developed an experimental system based on long-term gastrointestinal colonization of antibiotic-treated mice by the fungus *Candida albicans* coupled with serial faecal transplants from colonized to naive host. Clonal isolates harvested after 8 or 10 weekly serial passages (w8 or w10 strains) and it was found that the *Candida albicans* community gradually lost its ability to produce hyphae and showed a significantly increased intra-GI competitive fitness. W10 strains can also protect their new hosts against a variety of systemic infections. For instance, animals immunized with a w10 strain were protected from lethal doses of *A. fumigatus*, *Staphylococcus aureus,* or *Pseudomonas aeruginosa* [24].

### 4.3. Specific Gut Profile When HE Happened in Viral Hepatitis Cirrhotics

HE is a serious complication in viral hepatitis cirrhosis. When cirrhotics develop HE, there is a significantly increased *Haemophilus*, *Veillonella*, *Clostridium sensu stricto* or reduced *Fusobacterium*, *Megamonas*, and *Faecalibacterium*. Moreover, levels of *Bacteroides, Clostridium incertae sedis*, and *Clostridium XI* were significantly higher in survived patients; thus, they might be protective germs from the development of HE [29].

## 5. Clinical Implications of Gut Microbiota

### 5.1. Microbiota Index as a Model for Viral Hepatitis Cirrhotics

As there is a similar pattern in dysbiosis in cirrhotics, some investigators try to use microbiota indexes for diagnosis and prognostication in cirrhotics. One measure of the degree of dysbiosis in cirrhotics is the Cirrhosis Dysbiosis Ratio (CDR) reported by Bajaj [38]. This index is the ratio of (Lachnospiraceae + Ruminococcaceae + Veillonellaceae + Clostridiales Incertae sedis XIV)/(Bacteroidaceae + Enterobacteriaceae). A low CDR was associated with death and organ failures within 30 days and also linked to endotoxin, indicating a functional and ecologically plausible negative impact. Another index is Gut Dysbiosis Index (GDI) reported by Wang [42], GDI=∑OTUp/P − ∑OTUh/H, wherein OTUp or OTUh represents the patient-enriched or healthy-enriched OTUs identified by LEfSe. P and H represent the number of OTUs belonging to OTUp and OTUh, respectively. A higher GDI represents a more severe gut dysbiosis. The ratio of the relative abundance of fungal *Basidiomycota* vs that *of Ascomycota* can independently predict 90-day hospitalizations in cirrhotics regardless of cirrhosis severity and HE [40]. Moreover, the POD reported by Ren et al. [31] is based on the 30 bacterial OTUs markers that achieved powerful classification potential for distinguishing early HCC with the area under the curve (AUC) value of 76.80% from CHB cirrhotics. Recently, Bajaj et al. [43] found that adding a microbiota index significantly added to the MELD score in both RNA or DNA models to predict hospitalizations in cirrhotics.

### 5.2. Changing Profiles of Microbiota due to Medications for Viral Hepatitis Cirrhosis

Except for the routine use of antiviral treatments, HBV- or HCV-related cirrhotics take other medications such as proton-pump inhibitor (PPI), antibiotics, or lactulose. These drugs may affect the composition and function of gut microbiota.

Due to the decreased liver detoxification function and the formation of collateral circulation in cirrhotics, histamine and 5-hydroxytryptamine can enter the systemic circulation and stimulate gastric acid secretion. Therefore, PPIs were given to inhibit gastric acid secretion. Recently, Bajaj et al. [44] enrolled 15 decompensated cirrhotics (7 HCV-derived) to initiate PPI therapy and 15 decompensated cirrhotics (10 HCV-derived) to withdraw from PPI for 2 weeks. After the use of PPI for 2 weeks, the relative abundances of Lachnospiraceae and Ruminococcaceae were significantly reduced, while those of Streptococcaceae and Veillonellaceae were significantly increased. After PPI withdrawal, Porphyromonadaceae, Streptococcaceae, and Veillonellaceae were significantly reduced. From fungal perspective, PPI therapy did not significantly affect the fungal diversity in health controls or cirrhotics. It could be due to the fact that fungi are dependent on bacteria for nutrition and PPIs only affect certain species of bacteria without increasing or decreasing the total bacterial abundance. PPIs were associated with CDR, also further indicating that the effect of PPIs on gut microbiota may affect the prognosis of cirrhotics.

Antibiotics have antibacterial and germicidal effects. Neomycins, polymyxin B, paromomycin, norfloxacin, and rifaximin were commonly used. After the use of broad-spectrum antibiotics, the bacterial diversity in cirrhotics was reduced with changes in proportion of bacterial communities. In particular, rifaximin, a nonaminoglycoside antibiotic, is often used in cirrhotics accompanied by HE, accounting for a significant increase in Eubacteriaceae and *Propionibacterium*, as well as decreased Veillonellaceae, *Roseburia*, and *Blautia* [45]. The total fungi abundance was decreased after the use of broad-spectrum antibiotics. Sclerodermataceae, Dothideomycetes, and *Saccharomyces boulardii* were significantly reduced, while *Candida* was significantly increased. The ratio of *Basidiomycota/Ascomycete* was decreased, indicating that broad-spectrum antibiotics can destroy balanced bacterial and fungal communities and it is one of the major risk factors for fungal infections [40].

Lactulose is a nonabsorbable disaccharide, often used as an osmotic laxative to prevent HE, and has little effect on gut microbiota. HBV- or HCV-derived cirrhotics with HE used lactulose do not alter the alpha diversity of the gut microbiota, the abundance of bacteria that can produce ammonia, or other bacterial communities [46].

### 5.3. Therapeutic Intervention with Microbiota Modulation

FMT refers to the process of infusing faecal suspension from a healthy volunteer into the intestinal tract of patients with gastrointestinal diseases and is currently emerging as one of the more promising microbiota-modulating HBV- or HCV-related disease therapies. Bajaj et al. [47] applied FMT (faecal suspension enriched in Lachnospiraceae and Ruminococcaceae) to 10 cirrhosis patients (3 HCV-derived), after a 5-day broad-spectrum coverage regimen was used. Compared with 10 standard of care controls (4 HCV-derived), the result shows that, in advanced cirrhotics treated with lactulose and rifaximin, FMT restored antibiotic-associated disruption in microbial diversity and function, and there were significant fewer serious adverse events. The latest study showed that oral FMT capsules were also safe and well tolerated in HCV-derived cirrhotics with HE. These capsules enriched in Lachnospiraceae and Ruminococcaceae can improve duodenal mucosal diversity and duodenal antimicrobial peptide expression and reduced lipopolysaccharide-binding protein [48].

Probiotics are cultures of single or multiple microbes. The most common commercialized probiotics are lactose-fermenting *Lactobacilli* and *Bifidobacteria*. A meta-analysis made by Cai et al. [49], involving 826 minimal HE patients, indicated that probiotics had better efficacy in decreasing ammonia serum levels, endotoxemia levels, and hospitalization rates. Dhiman et al. [50] enrolled 130 cirrhosis patients including 21 HBV- or HCV-derived cirrhosis, and either probiotic VSL#3 or a placebo is given for 6 months. Compared with controls, daily intake of VSL#3 significantly reduced the risk of hospitalization for HE, MELD scores, inflammatory markers like TNF-*α*, IL-1b, and IL-6, plasma renin, aldosterone, blood ammonia, and indole levels.

Prebiotics are defined as nondigestible food ingredients or oligosaccharides until they reach the colon and can selectively stimulate the activity of one or numerous microbes. The most common prebiotics are inulin and galacto-oligosaccharides, often used to prevent HE through the acidification of feces, purgatory effects, or changing microbiota structure like increasing beneficial bacteria. The synbiotic is a compound of probiotics and prebiotics. Liu et al. [51] reported 97 cirrhotics (75 HBV or HCV-derived) and indicated that synbiotic treatment significantly increased the nonurease-producing *Lactobacillus* species and inhibited overgrowth of potentially pathogenic *Escherichia coli* and *Staphylococcal* species.

### 5.4. Potential Probiotics for the Adjuvant Treatment of Liver Injury

A recent study showed that *Lactobacillus rhamnosus GG* supplementation decreased hepatic bile acids (BA) by increasing intestinal FXR/FGF15 signaling pathway and enhances BA excretion, which prevents excessive BA-induced liver injury and fibrosis in mice [52]. *Bifidobacterium pseudocatenulatum LI09* and *Bifidobacterium catenulatum LI10* can alleviate the increase of M-CSF, MIP-1*α,* and MCP-1 in plasma and ameliorate the enrichment of the opportunistic pathogen *Parasutterella* and deplete the SCFA-producing bacteria *Anaerostipes*, *Coprococcus*, and *Clostridium XI* in mice [53]. *B. cereus* significantly improved serum ALT and cholinesterase levels in D-GalN-induced liver injury mice and modulated cytokine secretion [54]. Moreover, six strains of lactic acid bacteria with strong tolerance and adhesion ability decreased the expressions of AST, ALT, IL-6, and TNF-*α* factor in LPS/D-GalN- induced model group, suggesting the probiotic potential and pharmacological value of *L. paracasei* subspecies [55].

## 6. Conclusion

The gut microbiota has its own characteristics and is closely linked with viral hepatitis cirrhosis through the Gut-Liver Axis. Numerous studies have confirmed that there is a unique bacterial or fungal profile of viral hepatitis cirrhotics, strongly associated with specific metabolic status, immune responses, and changed endocrinological status when increased intestinal permeability, impaired gut barrier, SIBO, and bacterial translocation existed. The unique gut microbiota characteristics of viral hepatitis cirrhotics can be affected by different drugs. Though we have pushed forwards the use of FMT, probiotics, prebiotics, or synbiotics, there are some details that need to be further explored such as efficiency, safety, and drug abuse. In the near future, there is an emerging concern over the role of gut archaea and virome in viral hepatitis cirrhotics, implementation of FMT for severe clinical cases, microbiota as biomarkers for prognostication, and whether gut microbiota components or metabolites can be used as adjuvant immunotherapy for viral hepatitis cirrhosis and even assist the cure of viral hepatitis cirrhosis patients.

## Figures and Tables

**Table 1:** Microbiota in HBV/HCV cirrhotic patients vs controls.

Investigators	*N*	HBV/HCV-related cases (*n*)	Changed microbiota	
			Increased	Decreased
Chen et al. [32]	58	24 HBV-related	Firmicutes, *Veillonella*	Proteobacteria*, Neisseria*
			*Megasphaera*, *Dialister*	*Haemophilus*
			*Atopobium*, *Prevotella*	*SR1 genera incertae sedis*
Qin et al. [33]	181	99 HBV-related	Proteobacteria, Fusobacteria	*Bacteroidetes, Bacteroides*
			*Veillonella*, *Streptococcus*	*Eubacterium, Alistipes*
			*Clostridium*, *Prevotella*	
Xu et al. [34]	47	16 HBV-related	*B. dentium*	*B. catenulatum, B. infantis*
				*B. bifidum*
Ponziani et al. [35]	24	12 HCV-related	Proteobacteria, Staphylococcaceae	Methanobacteriaceae
			Veillonellaceae, Enterobacteriaceae	*Methanobrevibacter*
			Corynebacteriaceae, *Dialister*	
			*Staphylococcus,* Micrococcaceae	
			*Eubacterium, Enterococcus*	
Heidrich et al. [36]	145	38 HCV-related	Alloprevotella, *Lactobacillus*	*Clostridium IV, Bilophila*
			*Streptococcus, Veillonella*	*Mitsuokella, Victivallis*
			*Haemophilus, Akkermansia*	*Butyricimonas*
Aly et al. [37]	15	7 HCV-related	*Bacteroidetes, Prevotella*	*Ruminococcus, Clostridium*
			*Acinetobacter, Faecalibacterium*	*Parabacteroides*
			*Phascolarctobacterium, Veillonella*	
Bajaj et al. [38]	244	119 HCV-related	Staphylococcaceae, Enterococcaceae	Bacteroidaceae, Prevotellaceae
			Streptococcaceae	Clostridiales XIV
			Enterobacteriaceae	Ruminococcaceae*, Veillonellaceae*

*N* means the total number of patients in the study, which included healthy controls and those with HBV- or HCV-related cirrhosis.

## Abbreviations

ATP: Adenosine triphosphate
AUC: Area under the curve
BT: Bacterial translocation
BA: Bile acids
CDR: Cirrhosis dysbiosis ratio
CHB: Chronic hepatitis B
CHC: Chronic hepatitis C
DAMP: Damage-associated molecular patterns
FMT: Faecal microbial transplant
GDI: Gut dysbiosis index
HBV: Hepatitis B virus
HCC: Hepatocellular carcinoma
HE: Hepatic encephalopathy
LPS: Lipopolysaccharide
MELD: Model for end-stage liver disease
OTUs: Operational taxonomic units
PAMP: Pathogen-associated molecular patterns
PPI: Proton-pump inhibitor
SCFAs: Short-chain fatty acids
IBO: Small intestinal bacterial overgrowth
TLR: Toll-like receptor
TNF: Tumor necrosis factor.
